# Three-Stage Pooled Plasma Hepatitis C Virus RNA Testing for the Identification of Acute HCV Infections in At-Risk Populations

**DOI:** 10.1128/spectrum.02437-21

**Published:** 2022-05-02

**Authors:** Hsin-Yun Sun, Chieh Chiang, Sung-Hsi Huang, Wen-Jin Guo, Yu-Chung Chuang, Yi-Chia Huang, Chia-Jui Yang, Li-Hsin Su, Yi-Ting Chen, Yea-Wen Chen, Fu-Chiang Hsu, Shu-Yuan Ho, Wen-Chun Liu, Yi-Ching Su, Sui-Yuan Chang, Chin-Fu Hsiao, Chien-Ching Hung, Ming-Lung Yu

**Affiliations:** a Department of Internal Medicine, National Taiwan University Hospital, Taipei, Taiwan; b Department of Internal Medicine, National Taiwan University College of Medicine, Taipei, Taiwan; c Department of Mathematics, Tamkang University, New Taipei City, Taiwan; d Department of Internal Medicine, National Taiwan University Hospital Hsin-Chu Branch, Hsin-Chu, Taiwan; e Department of Tropical Medicine and Parasitology, National Taiwan University College of Medicine, Taipei, Taiwan; f Institute of Population Health Sciences, National Health Research Institutes, Zhunan, Taiwan; g Department of Internal Medicine, National Taiwan University Hospital Biomedical Park Branch, Hsin-Chu, Taiwan; h Department of Internal Medicine, Far Eastern Memorial Hospital, New Taipei City, Taiwan; i School of Medicine, National Yang Ming Chiao Tung University, Taipei, Taiwan; j Department of Laboratory Medicine, National Taiwan University Hospital, Taipei, Taiwan; k Department of Laboratory Medicine, National Taiwan University College of Medicine, Taipei, Taiwan; l Department of Clinical Laboratory Sciences and Medical Biotechnology, National Taiwan University College of Medicine, Taipei, Taiwan; m Department of Medical Research, China Medical University Hospital, Taichung, Taiwan; n China Medical University, Taichung, Taiwan; o Hepatobiliary Section, Department of Internal Medicine, Kaohsiung Medical University Hospital, Kaohsiung, Taiwan; p Hepatitis Research Center, College of Medicine, Kaohsiung Medical University, Kaohsiung, Taiwan; University of Arizona/Banner Health

**Keywords:** HIV infection, acute viral hepatitis, direct-acting antivirals, microelimination, sexually transmitted infection, test and treat

## Abstract

Timely diagnosis and treatment of hepatitis C virus (HCV) infection may prevent its transmission. We evaluated the performance and cost reductions of the pooled plasma HCV RNA testing strategy to identify acute HCV infections among people living with HIV (PLWH). PLWH with sexually transmitted infections, elevated aminotransferases within the past 6 months or past HCV infections (high-risk) and those without (low-risk) were enrolled prospectively. Participants underwent three-stage pooled plasma HCV RNA testing every 12 to 24 weeks until detection of HCV RNA or completion of a 48-week follow-up. The three-stage strategy combined 20 individual specimens into a stage 1 pool, 5 individual specimens from the stage 1 pool that tested positive for HCV RNA in the stage 2 mini-pool, followed by testing of individual specimens of the stage 2 mini-pool tested positive for HCV RNA. A simulation was constructed to investigate the cost reductions and pooled sensitivity and specificity under different combinations of HCV prevalence and pool/mini-pool sizes. Between June 25, 2019 and March 31, 2021, 32 cases of incident HCV viremia were identified in 760 high-risk PLWH that were enrolled 834 times, giving an incidence rate of 56.6 per 1000 person-years of follow-up (PYFU). No cases of HCV viremia were identified in 557 low-risk PLWH during a total of 269.2 PYFU. Simulation analysis suggested that this strategy could reduce HCV RNA testing cost by 50% to 86% with HCV viremia prevalence of 1% to 5% and various pooled sizes despite compromised pooled sensitivity. This pooled plasma HCV RNA testing strategy is cost-saving to identify acute HCV infections in high-risk populations with HCV viremia prevalence of 1% to 5%.

**IMPORTANCE** Our three-stage pooled plasma HCV RNA testing successfully identified HCV viremia in high-risk PLWH with a testing cost reduction of 84.5%. Simulation analysis offered detailed information regarding the selection of pool and mini-pool sizes in settings of different HCV epidemiology and the performance of HCV RNA testing to optimize the cost reduction.

## INTRODUCTION

Since 2000, hepatitis C virus (HCV) has rapidly spread mainly among HIV-positive men who have sex with men (MSM) in many developed countries (1). The risk factors for sexually transmitted HCV infection include concurrent sexually transmitted infections (STIs), high-risk sexual behaviors, use of mucosal administered drugs, and chemsex (2). Modeling suggests the high-risk individuals that compromise only 7% of the population contribute 94% of HCV infections, which could be markedly reduced by scaling-up treatments with direct-acting antivirals (DAAs) (3). Behavioral, not biological, factors drive the HCV epidemic among HIV-positive MSM, and the HCV treatment-as-prevention strategy could substantially lower the prevalence of HCV viremia in this population (4). In real-world settings, this strategy has effectively led to declines in HCV incidence and prevalence among people living with HIV (PLWH) (5–8).

To implement treatment-as-prevention for HCV, timely identification of individuals with acute HCV infections or HCV reinfections is crucial. A reliable diagnostic tool, such as HCV RNA testing, is essential given the delay of HCV seroconversion (9) and persistent HCV seropositivity after primary HCV infection. However, HCV RNA testing is limited by the cost incurred, especially in the high-risk populations who have risky exposures and often require repeated testing. Efforts need to be made to reduce HCV diagnostic and treatment costs to ensure the scale-up of HCV testing and treatment without restrictions.

Pooled testing combines individual specimens into a pooled specimen (10). The original and commonly used pooled testing is a two-stage strategy with binary responses. If a pooled specimen shows negative, all individual specimens are considered negative. If a pooled specimen is positive, at least one individual specimen would be positive. Thus, individual testing of each specimen is requested, a so-called two-stage pooling method. This method reduces the cost to screen many individuals for infectious diseases. The three-stage pooling method groups the initial pooled individual specimens (stage 1) into several mini-pooled specimens (stage 2) and tests the stage 2 mini-pooled specimens. Only the individual specimens in the positive stage 2 mini-pooled specimens are tested individually to determine which one is positive (stage 3). For example, if the initial pooled individual specimens (*n* = 20) are tested positive (stage 1), all the 20 individual specimens will be grouped into 4 mini-pooled specimens (5 individual specimens in each mini-pooled specimen, stage 2). Only the specimens in the positive mini-pooled specimens are tested individually to determine which one is positive (stage 3). The efficacy of the three-stage pooling method in the diagnosis of HCV viremia, however, is rarely assessed.

This study aimed to evaluate three-stage HCV RNA testing with the use of pooled specimens to identify acute or new HCV infections in both high-risk and low-risk populations. The impact of HCV prevalence and different combinations of pooled sizes of this strategy on the cost reductions and pooled sensitivity and specificity were also assessed.

## RESULTS

### Participants.

**FIG 1:**
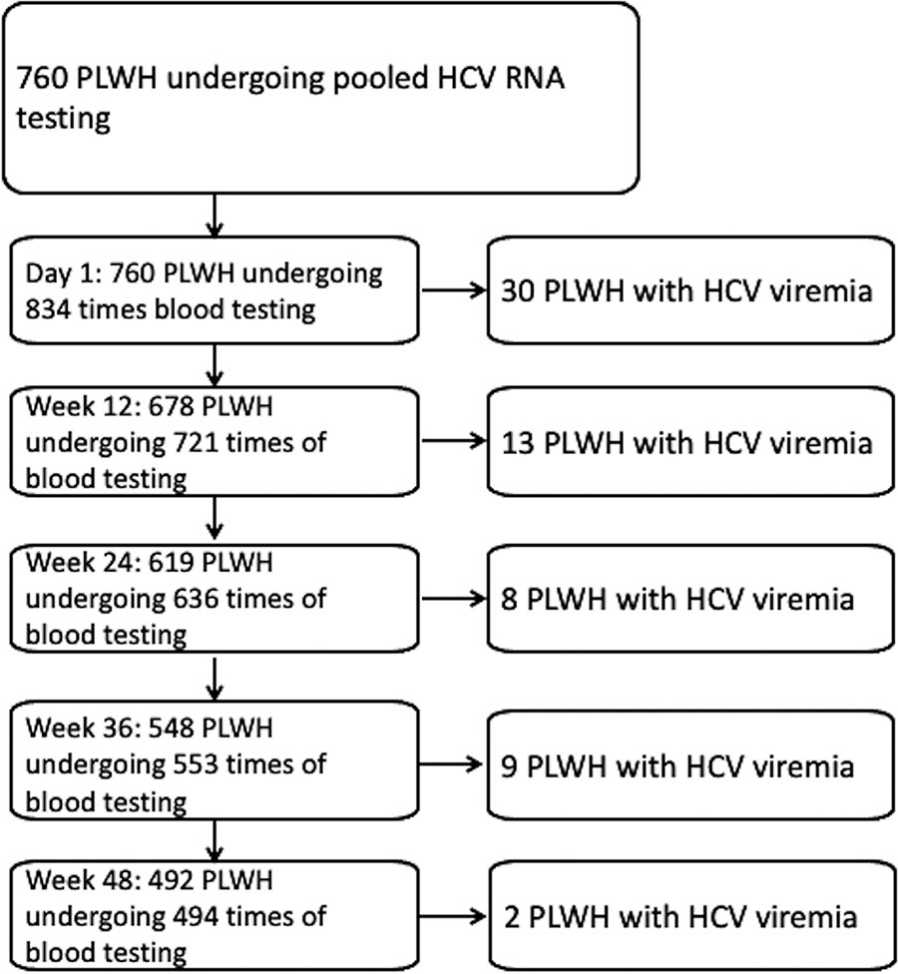
Study flow.

Between June 25, 2019 and March 31, 2021, 760 high-risk PLWH were recruited with 834 times of enrollment (Fig. 1). At enrollment (day 1), 30 PLWH tested positive for HCV viremia, 13 at week 12, 8 at week 24, 9 at week 36, and 2 at week 48. To estimate the incidence rate, the 30 individuals testing positive for HCV viremia on day 1 were excluded. During a total follow-up duration of 565.4 PYFU, 32 PLWH (4.2%, 32/760) developed HCV viremia, leading to an incidence rate of 56.6 per 1000 PYFU (95% CI 39.4 to 78.9).

The clinical characteristics of 760 high-risk PLWH (97.1% MSM), in which 9.7% were enrolled twice, are shown in Table 1. Anti-HCV was positive at enrollment at 27.0%. The inclusion criteria met in the high-risk group were STIs in 76.2%, HCV clearance by antivirals in 26.2% or spontaneously in 3.9%, and elevated aminotransferases in 6.8%. During the same study period, 557 low-risk PLWHs were enrolled. Compared with the high-risk group, they were older and more likely to be heterosexuals and have CD4 counts ≥200 cells/mm^3^, plasma HIV RNA <50 copies/mL, and positive hepatitis B virus surface antigen (HBsAg). None of the low-risk PLWH had HCV viremia by pooled plasma HCV RNA testing during a total of 269.2 PYFU.

**TABLE 1:** Comparisons of clinical characteristics of the PLWH at high risk and low risk for HCV infection who were enrolled for pooled HCV RNA testing

Variables	High-risk	Low-risk	*P* value
No. of PLWH, n	760	557	
Age, mean (SD), yrs	38.3 (8.6)	43.2 (11.3)	<0.001
Male, n (%)	759 (99.9)	525 (94.3)	<0.001
Enrolled once, n (%)	686 (90.3)	557 (100)	
Enrolled twice, n (%)	74 (9.7)	0 (0)	
Risk for HIV infection, n (%)			
MSM^*a*^	738 (97.1)	461 (82.8)	<0.001
Heterosexuals	10 (1.3)	68 (12.2)	
People who inject drugs	12 (1.6)	7 (1.3)	
At enrollment, n/N (%)			
cART in use	760 (100)	557 (100)	0.999
nNRTI	43 (5.7)	68 (12.2)	<0.001
PIs	8 (1.1)	18 (3.2)	0.005
INSTIs	722 (95.0)	481 (86.4)	<0.001
CD4 counts ≥200 cells/mm^3^	732/759 (96.4)	547/556 (98.4)	0.033
PVL <50 copies/mL	711/759 (93.7)	554 (99.5)	<0.001
Positive HBsAg, n/N (%)	63/759 (8.3)	67 (12.0)	0.025
Positive anti-HCV, n/N (%)	202/747 (27.0)	0 (0)	<0.001
Reasons for enrollment, n (%)			
Sexually transmitted infections	579 (76.2)	0 (0)	
Syphilis	360/481 (74.8)	0 (0)	
Achievement of sustained virologic response	199 (26.2)	0 (0)	
Spontaneous HCV clearance	30 (3.9)	0 (0)	
Abnormal liver function tests	52 (6.8)	0 (0)	
AST, median (IQR), IU/mL	47.5 (35.8–83.0)	NA	
ALT, median (IQR), UI/mL	78.5 (50.3–125.5)	NA	

a AST, aspartate transferase; ALT, alanine transferase; cART, combination antiretroviral therapy; HCV, hepatitis C virus; INSTI, integrase strand transfer inhibitor; IQR, interquartile range; MSM, men who have sex with men; NA, not applicable; nNRTI, nonnucleoside reverse transcriptase inhibitors; PI, protease inhibitor; PLWH, people living with HIV; PVL, plasma HIV RNA load; SD, standard deviation.

### Participants with HCV viremia by pooled HCV RNA testing.

Of 62 PLWH having HCV viremia at enrollment and during follow-up, the median plasma HCV RNA level was 6.4 log_10_ IU/mL (Table S2). Only 1 had HCV RNA <300 IU/mL (20 IU/mL by subsequent individual testing), which was detected because it was coincidentally included in a mini-pooled specimen with another specimen with HCV RNA of 70,000 IU/mL. This patient had positive anti-HCV at baseline; the test for HCV RNA turned positive during follow-up at week 24 and remained positive with an HCV RNA load of 37 IU/mL by individual HCV RNA testing 5 days after he tested positive by the pooled plasma HCV RNA testing. He subsequently achieved spontaneous HCV clearance. Twenty-seven (44.3%) had positive anti-HCV before enrollment. Among the other 34 PLWH with negative anti-HCV before enrollment, 15 (44.1%) remained HCV-seronegative when HCV viremia was identified.

DAAs were initiated in 47 PLWH with a median interval of 56 days (range 9 to 258) from detection of HCV viremia to treatment (Fig. S2). At the end of treatment (EOT), 1 patient interrupted DAA in the third month and remained positive for HCV RNA, 2 did not have data but achieved sustained viral response 12 weeks off-therapy (SVR12), and the remaining had undetectable HCV RNA. All 47 PLWH completed follow-up 12 weeks off-therapy, including 42 achieving SVR12, 4 having HCV viremia (3 with genotype switch and one HCV viremia at EOT), and 1 lost to follow-up. The main reason for the PLWH not initiating DAA was having received NHI-reimbursed DAA previously.

### Sensitivity and cost-saving analyses.

A total of 107 randomly selected stage 1 pooled specimens containing 11 individual specimens with detectable HCV RNA were tested by the pooled HCV RNA testing. All the negative individual specimens in these 107 pooled specimens were tested individually to confirm the results of the pooled testing. The results of individual testing were the same as those of the pooled testing. Instead of 3,540 individual HCV RNA tests, 550 were used to detect the 62 specimens with positive HCV RNA, and we were able to save 84.5% of the total testing cost required if all the collected specimens had been tested individually.

### Simulation of the three-stage pooled testing in different clinical settings.

**FIG 2:**
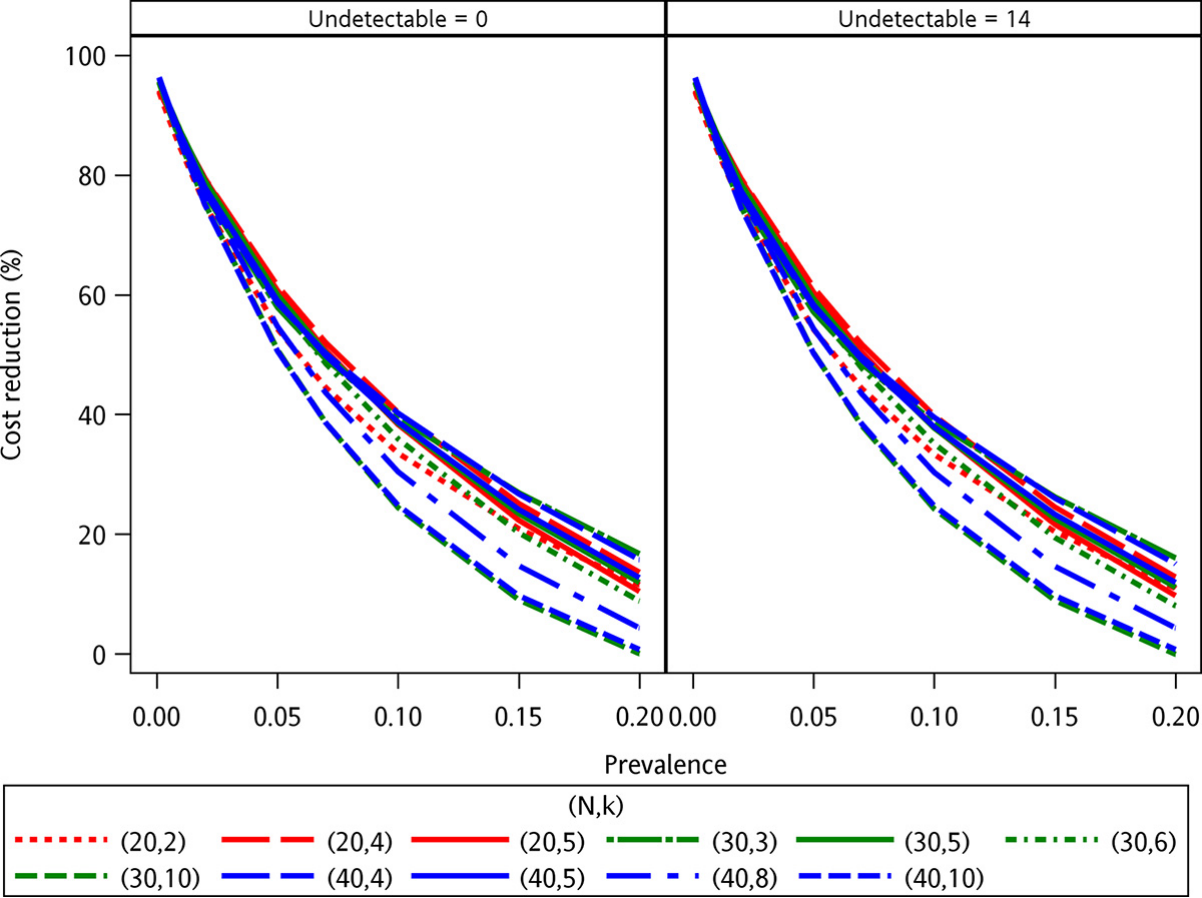
Cost reduction against prevalence with individual sensitivity of 98.94% and individual specificity of 99.99% by Cobas AmpliPrep HCV Test, v2.0, respectively. Given the detection limit of 15 IU/mL, the value of the undetectable individual specimen was assigned to 0 IU/mL (left), and that of the undetectable individual specimen is assigned to 14 IU/mL (right). With an HCV viremia prevalence at 1%, the cost of this strategy could be reduced by at least 86%. For an HCV viremia prevalence of 5%, this strategy could still provide cost reductions by at least 50%. When the HCV viremia prevalence was 10%, the cost reduction was less than 20% in all the settings. When the HCV prevalence was below 10%, the most efficient setting of this testing was (N = 20, k = 4), while the worst combination was (N = 40, k = 10). The most efficient setting was k = 5 (stage 2) regardless of any N (stage 1). When the HCV viremia prevalence was greater than 10%, the most efficient setting would be (N = 30, k = 3) while the worst combination would be (N = 30, k = 10). When the HCV viremia prevalence was 10%, the cost reduction was less than 20% in all the settings. The proposed testing costs more in the settings of (N = 30, k = 10) and (N = 40, k = 10) than individual testing for HCV viremia prevalence at 20%.

Based on the prevalence of HCV viremia of 1.8% (62/3540), the simulation of the three-stage pooled testing with the size of 10,000 specimens was analyzed. Fig. 2 demonstrates that the extent of cost reductions decreased as the HCV viremia prevalence increased. Table S3 summarizes the cost reductions by our strategy in the setting of different HCV viremia prevalence and pooled and mini-pooled sizes. The sensitivity of pooled HCV RNA testing decreased as the HCV prevalence became lower or the pooled size increased (Fig. S3). For the pooled specificity, all were better than the individual specificity regardless of any HCV viremia prevalence (Fig. S4).

In the setting of lower individual sensitivity and specificity, the performance of the pooled strategy regarding cost reductions against prevalence remained similar (Fig. S5), but that of the pooled sensitivity of 72% was considered poor when the individual sensitivity was 90% (Fig. S6). As for the pooled specificity, it decreased accordingly but was acceptable (Fig. S7).

**FIG 3:**
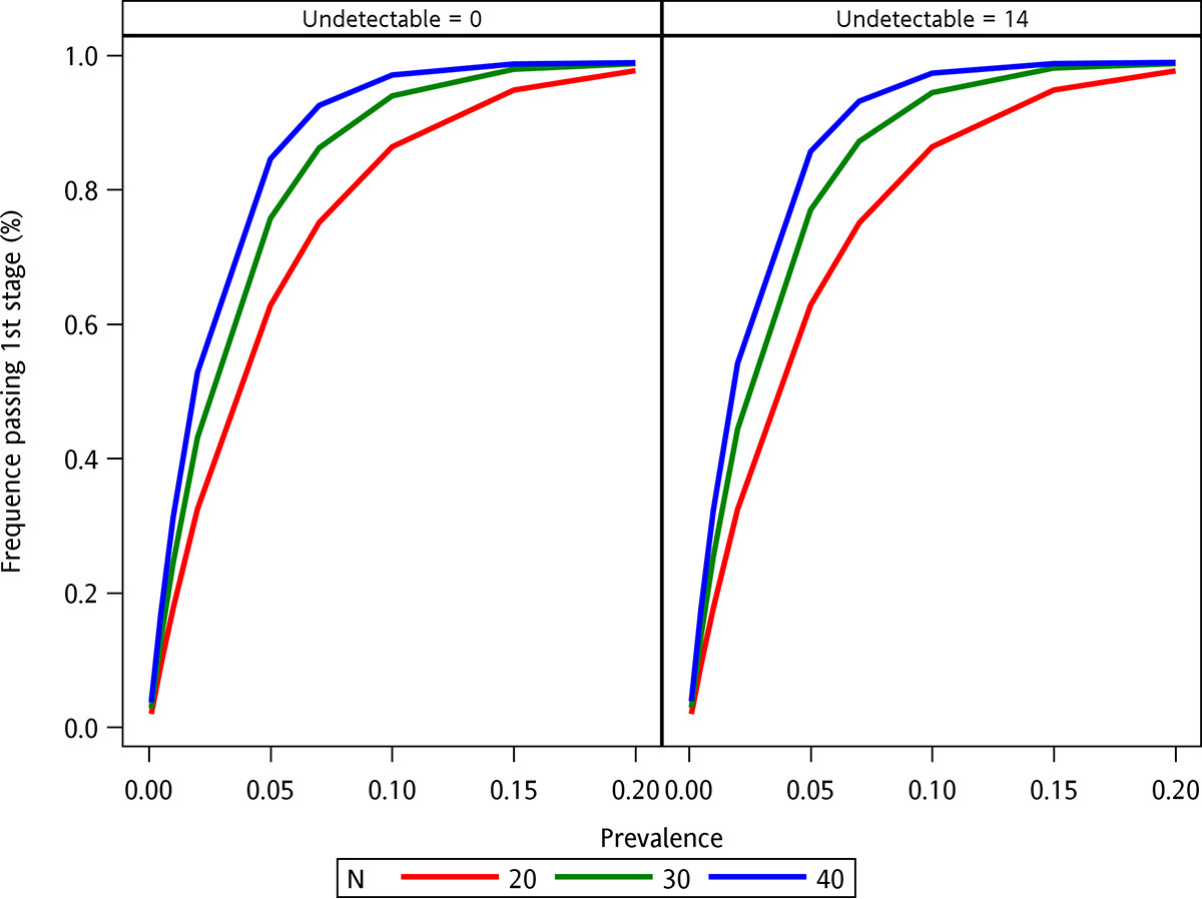
Rates of the three-stage pooled testing with positive HCV RNA results in the first stage against different prevalence. Given the detection limit of 15 IU/mL, the value of the undetectable individual specimen is assigned to 0 IU/mL (left), and that of the undetectable individual specimen is assigned to 14 IU/mL (right). When the HCV viremia prevalence was greater than 10%, the positive HCV RNA rate in the first stage was greater than 80% and 98% if the pooled size was 40. In this situation, it was suggested to carry out a two-stage pooled testing directly because 98% of testing in the first stage would be tested positive for HCV RNA. However, if the pooled size was 20, the positive rate was 87% meaning a three-stage pooled strategy could still save a cost of 13% more than a two-stage pooled strategy.

Fig. 3 compares the efficacy between three-stage and two-stage pooled testing. When the HCV viremia prevalence was greater than 10%, the positive rate was 87% if the pooled size was 20, meaning the three-stage pooled strategy could still save cost by 13% more than the two-stage pooled strategy.

## DISCUSSION

In the at-risk populations for acute HCV infections, we demonstrated that regular three-stage pooled plasma HCV RNA testing can be a feasible and cost-saving strategy to monitor high-risk populations for acute HCV infections. By this strategy, 84.5% of the direct testing cost could be saved. By simulation, when the HCV viremia prevalence ranges from 1% to 5%, the cost would be reduced by 50 to 86% with variable pooled combinations (Fig. 2). Despite the compromised pooled sensitivity (Fig. S3), regular HCV testing with small, pooled sizes might overcome this limitation given the high HCV RNA loads in the setting of acute HCV infections while the pooled specificity is less affected (Fig. S4). Our simulation demonstrated the performance of this strategy in various clinical scenarios regarding different individual sensitivities and specificities to optimize its performance (Fig. S5 to S7). With HCV prevalence greater than 10%, the performance of the three-stage strategy remained better than that of the two-stage strategy by a cost reduction of 13% only when the pooled size was 20 (Fig. 3). Our study suggests that this strategy could be tailored to different local HCV epidemiology and risk populations (Table S3).

We demonstrated an efficient and cost-saving strategy to scale up the early diagnosis of HCV infections and link these individuals to receive DAAs. Among the 34 PLWH with negative anti-HCV before enrollment, 44.1% remained HCV-seronegative when HCV viremia occurred. HCV RNA turning from negative to positive was documented subsequently in 51 (82.3%) of the 62 PLWH testing HCV RNA-positive, indicating that this strategy is effective in the early identification of acute HCV infections.

The optimal testing frequency to detect acute HCV infections remains undetermined. The US guidelines recommend anti-HCV testing annually or as indicated by risk exposure (11). European AIDS Clinical Society (EACS) guidelines suggest HCV screening based on risk behaviors and local epidemiology (12). For HIV-positive MSM with HCV clearance after antivirals, testing for HCV reinfections every 3 to 6 months is recommended (13). Given the missed diagnosis of HCV reinfections by either aminotransferase- or syphilis-guided testing strategy (14), we employed risk-based enrollment and regular (every 3 months) HCV RNA testing in this study, which successfully identified the populations at risk for acute HCV infections. With our strategy, more PLWH with chronic and acute HCV infections could be diagnosed at lower costs. With the implementation of the HCV treatment-as-prevention strategy, the disease burden and onward transmission of HCV infection could be reduced. As shown in the studies from several European countries (5–8), the incidence and prevalence of HCV viremia in PLWH may decline with an improvement in access to testing and the lifting of restrictions on treatment eligibility.

After the diagnosis of recently acquired HCV infections in PLWH, EACS guidelines suggest HCV treatment when PLWH had a reduction of <2 log_10_ in HCV RNA load 4 weeks later (15). However, without HCV treatment and modifications of their sexual behaviors, these PLWHs were highly infectious given their high HCV RNA loads (median, 6.4 log_10_ IU/mL; range, 1.3 to 8.5 log_10_ IU/mL) at diagnosis and their viral loads remained high (median, 6.1 log_10_ IU/mL; range, 1.6 to 8 log_10_ IU/mL) in their repeated HCV RNA testing after a median follow-up duration of 28 days (IQR, 13.0 to 84.0). Due to the uncertainty in behavioral modifications and a lower rate of spontaneous HCV clearance in PLWH (16), rapid initiation of HCV treatment should be a better strategy to reduce the risk of onward HCV transmission, which is similar to the observed zero risk of HIV transmission in virally suppressed PLWH (17, 18).

The study has several limitations. First, given the compromised sensitivity after the combination of 20 specimens together, we might miss the individual specimens with HCV RNA <300 IU/mL (Table S1). More investigations are needed to better define the lower levels of HCV RNA detection with this pooling strategy of different pool sizes. However, due to the unavailability of sufficient clinical samples with a wide range of HCV RNA loads, particularly those with HCV RNA loads near the limit of detection, the impact of the number of samples with weak positives in a larger testing population on positive HCV RNA results by pooled plasma HCV RNA testing could only be evaluated by the simulation analysis with the size of 10,000 specimens and the detection limit of 15 IU/mL by individual HCV RNA testing and the value of the undetectable individual specimens were assigned to 0 IU/mL and 14 IU/mL, respectively (Fig. S2). Second, the effectiveness of a pooling strategy depends largely on the pretest probability of HCV infection (19). As shown in Fig. 2, the suitable HCV prevalence for this strategy to be most cost-effective ranges between 1% and 5%, leading to cost reductions of 50% to 86%. Third, we did not include the costs of all consumables required for HCV RNA testing and the time spent by laboratory staff in the estimation of cost reductions. Fourth, how the implementation of pooled HCV RNA testing changes the incidence and prevalence of acute HCV infections warrants more investigations.

In conclusion, regular three-stage pooled HCV RNA testing is cost-saving and feasible to timely identify new HCV infections in high-risk populations and prompts the rapid initiation of HCV treatment, which could potentially reduce onward transmission of HCV through sexual contacts or contaminated body fluid or blood.

## MATERIALS AND METHODS

### Study population and setting.

HCV testing, including anti-HCV antibody or HCV RNA testing, is recommended for PLWH once annually or clinically indicated by the national HIV treatment guidelines in Taiwan. Increasing trends of recently acquired HCV infections had been noted in Taiwan from 1994 to 2018, with an incidence rate of 28.10 per 1000 person-years of follow-up (PYFU) in 2018 (20, 21), and that of HCV reinfection of 82 per 1000 PYFU (14). Syphilis was significantly associated with HCV infections in all these studies (14, 20, 21). In Taiwan, DAAs are reimbursed by the National Health Insurance (NHI) after 2017 (22, 23). Hepatologists and HIV-treating physicians are responsible for HCV treatments in eligible PLWH. The program did not reimburse DAA retreatment for those who had received NHI-reimbursed DAAs during the conduct of this study.

### Participants.

The study recruited PLWH receiving medical care at a university hospital that provides primary and tertiary patient care in northern Taiwan. The high-risk populations included PLWH with STIs, elevated aminotransferases within the past 6 months, or spontaneous HCV clearance or achievement of sustained virologic response (SVR) by antivirals. Individuals with previously documented HCV viremia but untreated were excluded. The low-risk populations were PLWH with negative anti-HCV and no STIs or elevated aminotransferases in the past 6 months.

The high-risk group underwent the pooled plasma HCV RNA testing every 12 weeks or clinically indicated, and the low-risk group every 24 weeks (13). Participants were followed until the detection of HCV RNA, loss to follow-up, death, or completion of 48-week follow-up. Participants with newly identified HCV viremia by this strategy were advised to return to the clinic to undergo further blood testing for assessment of eligibility for NHI-reimbursed DAAs. The assessment required included HCV RNA load, HCV genotyping, serum albumin, total and direct bilirubin, aminotransferases, prothrombin time, and hepatitis B surface antigen.

Repeated participation in the pooled HCV RNA testing was allowed for those testing negative for HCV RNA and having recurrent STIs or elevated aminotransferases after completing a 48-week follow-up, or those having tested positive for HCV RNA with viral clearance either spontaneously or after DAA treatment. To calculate the incidence rate of acute HCV infection, participants testing positive for HCV RNA at enrollment were not considered to have incident HCV infections but were included in the analysis of the performance of three-stage pooled plasma HCV RNA testing (Table 1).

Given the incidence of recently acquired HCV infection of 3.4 to 8.4% (20, 21), 20 individual specimens (plasma of 50 microliters from each specimen, the pooled size, denoted by N = 20) were combined into a pooled specimen for HCV RNA testing (stage 1). All the 20 individual specimens would be considered free of HCV if the pooled specimen tested negative for HCV RNA, which took approximately 2.7 h to get the result. For any positive pooled specimens, every 5 (plasma of 150 microliters from each specimen, the mini-pooled size, denoted by k = 5) of the 20 individual specimens were combined into a mini-pooled specimen for testing (stage 2). For a positive mini-pooled specimen, each of the 5 specimens was retested individually to identify the one with HCV RNA (stage 3) (Fig. S1). It took approximately 9 h to go through stages 1 to 3.

We assessed the feasibility and performance of this strategy in real-world settings, and reductions of direct cost in HCV RNA testing by the strategy were estimated. This prospective study was approved by the Research Ethics Committee of the hospital (201904086RIPB), and all participants gave written informed consent. Given the difficulties in statistical estimations for multistage pooled testing (24), a computational simulation was performed to characterize this three-stage pooling strategy. Because the sensitivity of a pooling strategy is known to be decreased (25, 26), the impact of the compromised pooled sensitivity was also evaluated.

### Laboratory investigations.

HCV RNA was determined using Cobas AmpliPrep HCV Test, v2.0 (Roche, USA) before and Cobas 6800 (Roche, USA) after September 2020 (the detection limit, 15 IU/mL) and a fourth-generation enzyme immunoassay (Dia.Pro Diagnostic Bioprobes Srl. Italy) (21). The detection limit of stage 1 pooled specimens was arbitrarily estimated at 300 IU/mL after the pooling of 20 individual specimens. However, the exactly lower level of detection by this pooled plasma HCV RNA testing might not be clearly defined. We performed the analytical sensitivity of pooled samples using 20 individual positive clinical samples (5 samples with HCV RNA loads <300 IU/mL and 15 with loads between 300 to 1000 IU/mL), and the detailed results are presented in supplemental materials (Table S1). We also did a sensitivity analysis to compare the detection rates by individual and pooled testing for the 107 stage 1 pools (consisting of a total of 2,140 individual specimens). Anti-HCV antibodies of the specimens with detectable HCV RNA were determined individually if the participants had negative anti-HCV before they tested positive by pooled HCV RNA testing.

### Statistical analysis.

Categorical variables were compared using *X*^2^ or Fisher’s exact test, and noncategorical variables were Student's *t* test or Mann-Whitney U test by Statistical Program for Social Sciences (SPSS Statistics Version 21, IBM Corp., Armonk, New York). Text S1 describes the methods for the simulation of this three-stage pooled strategy to assess the impact of different combinations of pooled sizes in the setting of individual specimens with different undetectable values, individual sensitivities and specificities of HCV RNA testing, and the prevalence of HCV viremia ranging from 0.1% to 20% on the cost reductions, and pooled sensitivity and specificity, and provide a computational program based on statistical software R.

### Data availability.

Deidentified participant-level will be available on publication of the study. Requests for data should be sent to fish6069@gmail.com and, on review of the proposed protocol and signing of a data-sharing agreement, the data will be made available. The protocol and consent form will also be available by email request.
