# Supplementary material for: Three-Stage Pooled Plasma Hepatitis C Virus RNA Testing for the Identification of Acute HCV Infections in At-Risk Populations
Source: Microbiol Spectr. 2022 May 2;10(3):e02437-21. doi: 10.1128/spectrum.02437-21 (PMC9241589; doi:10.1128/spectrum.02437-21)

Supplementary Materials: Three-stage Pooled-Plasma HCV RNA Testing for the Identification of Acute HCV Infections in At-Risk Populations

Methods

Simulation analysis

Let N be the pooled size of the first stage. Let X_i be the number of HCV RNA for a specimen, $i=1,...,N$. At the first stage, let $\phi_N=1$ if $\sum_{i=1}^{N} X_j / N \geq c$, otherwise, 0. Here c is the detectable limit. Also, let k be the pooled size of each subgroup at the second stage, let $\phi_{k,j}=1$ if $\sum_{i=(j-1)k+1}^{jk} X_i / k \geq c$, otherwise, 0, for $j=1,...,N/k$.

Then, the required tests for the proposed three-stage pooled testing was

$$T=1+\left[\phi_{Se,N}\phi_N+\left(1-\phi_{Sp,N}\right)\left(1-\phi_N\right)\right]\left\{\frac{N}{k}+k\sum_{j=1}^{N/k}\left[\phi_{Se,k,j}\phi_{k,j}+\left(1-\phi_{Sp,k,j}\right)\left(1-\phi_{k,j}\right)\right]\right\},$$

where $\phi_{Se,N}=1$ and $\phi_{Se,k,j}=1$ with sensitivity, say Se, and $\phi_{Sp,N}=1$ and $\phi_{Sp,k,j}=1$ with specificity, say Sp.

It was noted that ϕ_N and $\phi_{k,j}$'s were not independent. For example, with $N=10$, $k=5$ and $\phi_{10}=1$, we must have either $\phi_{5,1}=1$ or $\phi_{5,2}=1$. Also, if $\phi_{5,1}=0$, we must have $\phi_{5,2}=1$. Therefore, the dependence led to the difficulty of deriving derive the statistical characteristics. Alternatively, a simulation of the three-stage pooled testing was applied.

Simulation of the three-stage pooled testing

Step 1: Generate N indicators (detectable or undetectable) from a Bernoulli distribution with prevalence, say P. If the indicator was 0, then the specimen was denoted by undetectable. In this case, we still assigned a value to this undetectable specimen. Else if the indicator was detectable, then we would randomly select a value from the real detectable database.

Step 2: The simulated three-stage pooled testing was performed; that is, the N specimens were pooled to test. The testing was continued to the second stage with probability of Se if $\sum_{i=1}^{N} X_i / N \geq c$; otherwise, the testing was continued to the second stage with probability of $1-Sp$; otherwise, the testing was stopped and the N specimens were considered as undetectable.

Step 3: At the second stage, every k specimens were pooled to test. The testing was continued to the third stage with probability of Se for each subgroup if $\sum_{i=(j-1)k+1}^{jk} X_i / k \geq c$ for $j = 1, \ldots, N/k$; otherwise, the testing was continued to the third stage with probability of $1-Sp$; otherwise, the subgroup was stopped to test and this k specimens were considered as undetectable.

Step 4: At the third stage, every specimen was tested individually. If the X was greater than c, then the specimen was denoted by detectable with probability of Se; else if the X was less than c, then the specimen was denoted by detectable with probability of $1-Sp$; otherwise, undetectable.

The next section is the R code of the proposed 3-stage pooled strategy. This R code allows user to specify the prevalence, individual sensitivity, individual specificity, N, and k. An additional csv file is required to provide a sample of value of detectable responses. The first row in the csv file is the variable name (can be arbitrary). The following figure is an example of the content:

	A	B
1	HCV_RNA	
2	8450000	
3	20150000	
4	9450000	
5	1380000	
6	149000	
7	10650000	
8	1875000	
9	11850000	
10	1685000	
11	6700000	

While an example of the output is as following figure:

```
$`Procedure Size`
Prevalence          N          k
     0.018     20.000      5.000

$`Number of tests in one procedure`
Mean   SD
 3.9  4.5

$Estimates
  Cost_Reduction Test_Sensitivity Test_Specificity       Prevalence
          0.8069           0.9551           1.0000           0.0172
```

Supplementary R code

a <- read.csv("E:/AAA.csv")   #Pathway of the input data

UDTV <- 14   #Value of undetectable viral load

```
Se <- 0.9894                          #Individual sensitivity

Sp <- 1.0000                          #Individual specificity

N <- 20                               #Size of the first stage

k <- 5                                #Size of the second stage

p <- 0.018                            #Prevalence rate

#==================================================================================#

MyCfunc <- function(DT,UDTV,Se,Sp,N,k,p){

 Ng <- 100              #Number of procedures

 NSS <- Ng*N

 a1 <- rbinom(NSS,1,p)

 a2 <- array(0,NSS)

 a3 <- a1

 for(i in 1:NSS){

  if(a1[i]==1){

    a2[i] <- sample(DT, 1, replace = TRUE)

  }else{

    a2[i] <- UDTV

  }

 }

 kg <- N/k

 c <- 0                 #Number of Detection

 p15 <- 0               #Number of Positive in the 3rd Tier
```

```
mytemp <- NULL
for(i in 1:Ng){
  L20 <- 1+N*(i-1)
  U20 <- N*i
  c <- c + 1
  if((sum(a2[L20:U20])/N) >= 15){
    #First Tier
    tSe <- rbinom(1,1,Se)
    if(tSe==1){
      for(j in 1:kg){
        Lk <- L20+k*(j-1)
        Uk <- L20-1+k*j
        c <- c + 1
        if((sum(a2[Lk:Uk])/k) >= 15){
          #Second Tier
          tSe <- rbinom(1,1,Se)
          if(tSe==1){
            c <- c + k
            for(l in Lk:Uk){
              if(a2[l] >= 15){
                #Third Tier
                tSe <- rbinom(1,1,Se)
```

```
        if(tSe==1){
          p15 <- p15 + 1
          a3[l] <- 1
        }else{
          a3[l] <- 0
        }
      }else{
        tSp <- rbinom(1,1,Sp)
        if(tSp==1){
          a3[l] <- 0
        }else{
          p15 <- p15 + 1
          a3[l] <- 1
        }
      }
    }
  }else{
    a3[Lk:Uk] <- 0
  }
}else{
  #Second Tier
  tSp <- rbinom(1,1,Sp)
```

```
if(tSp==1){
  a3[Lk:Uk] <- 0
}else{
  c <- c + k
  for(l in Lk:Uk){
    if(a2[l] >= 15){
      #Third Tier
      tSe <- rbinom(1,1,Se)
      if(tSe==1){
        p15 <- p15 + 1
        a3[l] <- 1
      }else{
        a3[l] <- 0
      }
    }else{
      tSp <- rbinom(1,1,Sp)
      if(tSp==1){
        a3[l] <- 0
      }else{
        p15 <- p15 + 1
        a3[l] <- 1
      }
```

```
          }
        }
      }
    }
  }
 }else{
  a3[L20:U20] <- 0
 }
}else{
 #First Tier
 tSp <- rbinom(1,1,Sp)
 if(tSp==1){
  a3[L20:U20] <- 0
 }else{
  for(j in 1:kg){
   Lk <- L20+k*(j-1)
   Uk <- L20-1+k*j
   c <- c + 1
   if((sum(a2[Lk:Uk])/k) >= 15){
    #Second Tier
    tSe <- rbinom(1,1,Se)
    if(tSe==1){
```

```
  c <- c + k
  for(l in Lk:Uk){
    if(a2[l] >= 15){
      #Third Tier
      tSe <- rbinom(1,1,Se)
      if(tSe==1){
        p15 <- p15 + 1
        a3[l] <- 1
      }else{
        a3[l] <- 0
      }
    }else{
      tSp <- rbinom(1,1,Sp)
      if(tSp==1){
        a3[l] <- 0
      }else{
        p15 <- p15 + 1
        a3[l] <- 1
      }
    }
  }
}else{
```

```
    a3[Lk:Uk] <- 0
  }
}else{
  #Second Tier
  tSp <- rbinom(1,1,Sp)
  if(tSp==1){
    a3[Lk:Uk] <- 0
  }else{
    c <- c + k
    for(l in Lk:Uk){
      if(a2[l] >= 15){
        #Third Tier
        tSe <- rbinom(1,1,Se)
        if(tSe==1){
          p15 <- p15 + 1
          a3[l] <- 1
        }else{
          a3[l] <- 0
        }
      }else{
        tSp <- rbinom(1,1,Sp)
        if(tSp==1){
```

```
        a3[l] <- 0
       }else{
        p15 <- p15 + 1
        a3[l] <- 1
       }
      }
     }
    }
   }
  }
 }
 }
 mytemp <- cbind(mytemp,c)
 c <- 0
}
TP <- 0                 #True Positive
FN <- 0                 #False Negative
TN <- 0                 #True Negative
FP <- 0                 #False Positive
for(i in 1:NSS){
 if(a3[i]<a1[i]){
  FN <- FN + 1
```

```
   }else if(a3[i]>a1[i]){
     FP <- FP + 1
   }else if(a3[i]==1 && a1[i]==1){
     TP <- TP + 1
   }else if(a3[i]==0 && a1[i]==0){
     TN <- TN + 1
   }
  }
  ESe <- TP/(TP+FN)              #Empirical Sensitivity
  ESp <- TN/(TN+FP)              #Empirical Specificity
  temp <- mean(mytemp)
  c(temp, sd(mytemp), 1-temp/N, ESe, ESp, p15/NSS)
}
#==================================================================================#
MyCfunc2 <- function(DT,UDTV,Se,Sp,N,k,p){
  if(N%%k==0){
  Ns <- 10000       #Number of simulations
  mytable <- NULL
  for(tt in 1:Ns){
   mytable <- rbind(mytable,MyCfunc(DT,UDTV,Se,Sp,N,k,p))
  }
  mymean <- apply(mytable,2,mean)
```

```
  Mytemp <- c(p,N,k,round(mymean[1:2],1),round(mymean[3:6],4))
  names(Mytemp) <- c("Prevalence","N","k","Mean","SD","Cost_Reduction",
                 "Test_Sensitivity","Test_Specificity","Prevalence")
  Mytemp2 <- list(Mytemp[1:3], Mytemp[4:5], Mytemp[6:9])
  names(Mytemp2) <- c("Procedure Size",
                  "Number of tests in one procedure",
                  "Estimates")
  Mytemp2
  }else{print("ERROR!N must be exactly divisible by k.")}
}
#===================================================================================#
DT <- a[,1]
MyCfunc2(DT,UDTV,Se,Sp,N,k,p)
```

Table S1. The analytical sensitivity of pooled samples. Twenty individual positive clinical samples with 25% of the positive samples near the limit of detection of the assay (5 samples with HCV RNA loads <300 IU/mL and 15 with loads between 300 to 1000 IU/mL) were selected to determine the analytical sensitivity of pooled samples. Each positive sample were pooled with other 19 negative samples as described in Methods. The determination of HCV RNA loads by pooled testing was repeated twice, and the results are shown in the following table. For the 5 samples with HCV RNA loads <300 IU/mL, the sensitivity of pooled testing ranges 0-50%. For the 15 samples with HCV RNA loads of 300-1000 IU/mL, the sensitivity of pooled testing is 86.7% (13/15). The two negative results came from samples of 313 and 367 IU/ml, respectively.

Patient Number	Original HCV RNA loads by individual testing		The 1st HCV RNA loads after pooled testing			The 2nd HCV RNA loads after pooled testing		
	IU/mL	log_{10} IU/mL	IU/mL	log_{10} IU/mL	Differences*	IU/mL	log_{10} IU/mL	Differences*
1	276	2.44				440	2.64	0.20
2	518	2.71	394	2.60	-0.12	628	2.80	0.08
3	526	2.72	414	2.62	-0.10	466	2.67	-0.05
4	367	2.56						
5	435	2.64	746	2.87	0.23	684	2.84	0.20
6	175	2.24				362	2.56	0.32
7	928	2.97	1716	3.23	0.27	900	2.95	-0.01
8	556	2.75	534	2.73	-0.02	614	2.79	0.04
9	313	2.50						
10	356	2.55	408	2.61	0.06	548	2.74	0.19
11	265	2.42						
12	317	2.50	428	2.63	0.13	460	2.66	0.16

13	139	2.14	808	2.91	0.76			
14	181	2.26				422	2.63	0.37
15	595	2.77	878	2.94	0.17	766	2.88	0.11
16	964	2.98	1278	3.11	0.12	746	2.87	-0.11
17	616	2.79	356	2.55	-0.24	442	2.65	-0.14
18	734	2.87	336	2.53	-0.34	1024	3.01	0.14
19	870	2.94	776	2.89	-0.05	936	2.97	0.03
20	338	2.53	806	2.91	0.38	752	2.88	0.35

Footnote: The grey and black bars mean no detection of HCV RNA.

*Differences in HCV RAN loads ($\log_{10}$ IU/mL) by individual or pooled testing

Table S2. Clinical characteristics of 62 people living with HIV who tested positive for HCV viremia in the pooled HCV study

Variables	Data
Participant number	62
Participants with HCV viremia at the first determination, n (%)	30 (48.4)
Participants with documented acute HCV infection*	51 (82.3)
Interval from negative to positive HCV viremia, median (IQR), days	91 (81-105)
Age, median (IQR), years	33.1 (29.6-40.0)
Men, n (%)	62 (100)
HIV transmission risk, n (%)	
Men who have sex with men	61 (98.4)
Heterosexuals	0 (0)
Injection drug users	1 (1.6)
At enrollment, n/N (%)	
CD4 count ≥200 cells/mm^3	57/61 (93.4)
Plasma HIV RNA load <50 copies/mL	53/62 (85.5)
Positive anti-HCV before enrollment	27/61 (44.3)
Having received HCV treatment (both IFN-based and DAA)	20/62 (32.3)
NHI-reimbursed DAA treatment	12/20 (60.0)
Reasons for enrollment, n (%)	
Occurrence of sexually transmitted infections	46 (74.2)

Achievement of sustained virologic response	18 (29.0)
Development of spontaneous clearance	4 (6.5)
Abnormal liver function	11 (17.7)
At the time of positive pooled HCV RNA	
Plasma HCV RNA load, median (range), $\log_{10}$ IU/mL	6.4 (1.3-8.5)
Participants with negative anti-HCV, n (%)	15 (24.2)
Negative anti-HCV in participants with negative anti-HCV before enrolment	15/34 (44.1)
Participants initiating DAA treatment, n (%)	47 (75.8)
Interval from diagnosis to treatment, median (range), days	56 (9-258)
DAA treatment reaching EOT, n (%)	47 (100)
Detectable HCV RNA at EOT	1/47 (2.1)
Undetectable HCV RNA at EOT	44/47 (93.6)
No data at EOT	2/47 (4.3)
Follow-up reaching 12 weeks after EOT, n (%)	47 (75.8)
Undetectable HCV RNA 12 weeks after EOT (SVR12)	42 (89.4)
Positive HCV RNA 12 weeks after EOT	4** (8.5)
Loss to follow-up 12 weeks after EOT	1 (2.1)
Participants not receiving DAAs, n (%)	15 (24.2)
History of previous NHI-reimbursed DAA treatment	12/15 (70.6)
Spontaneous clearance of HCV infection	1/15 (5.9)

Loss to follow-up	1/15 (5.9)
Incarceration	1/15 (5.9)

*Acute HCV infection was defined as HCV viraemia identified in individuals without HCV viremia within the past one year

**Three participants with HCV viraemia 12 weeks after EOT experienced HCV genotype switch after off-DAA for 3 months, and the 4th PLWH was the one who had detectable HCV RNA at EOT.

Abbreviations: DAA, direct-acting antiviral; EOT, end of treatment; HCV, hepatitis C virus; IFN, interferon; SVR12, sustained virologic response 12 weeks off-treatment.

Table S3. Averaged results from 10,000 times simulation study with individual sensitivity of 0.9894 and individual specificity of 0.9999 in the setting of different HCV viremia prevalence, and pooled and mini-pooled sizes.

			Number of tests		Cost	Three-stage	pooled strategy
Prevalence	N	k	Mean	SD	reduction	Sensitivity	Specificity
0.001	20	2	1.2	1.6	0.9384	0.9502	1.0000
		4	1.2	1.2	0.9413	0.9499	1.0000
		5	1.2	1.2	0.9413	0.9500	1.0000
0.001	30	3	1.4	2.1	0.9547	0.9136	1.0000
		5	1.3	1.8	0.9565	0.9137	1.0000
		6	1.3	1.8	0.9565	0.9138	1.0000
		10	1.4	2.1	0.9547	0.9138	1.0000
0.001	40	4	1.5	2.6	0.9621	0.9141	1.0000
		5	1.5	2.5	0.9630	0.9141	1.0000
		8	1.5	2.5	0.9631	0.9140	1.0000
		10	1.5	2.7	0.9621	0.9144	1.0000
0.005	20	2	2.1	3.5	0.8940	0.9500	1.0000
		4	1.8	2.7	0.9077	0.9502	1.0000
		5	1.8	2.7	0.9077	0.9503	1.0000
0.005	30	3	2.7	4.5	0.9092	0.9178	1.0000
		5	2.5	3.8	0.9176	0.9180	1.0000
		6	2.5	3.9	0.9174	0.9180	1.0000

		10	2.8	4.6	0.9082	0.9184	1.0000
0.005	40	4	3.4	5.4	0.9140	0.9193	1.0000
		5	3.3	5.1	0.9179	0.9195	1.0000
		8	3.3	5.2	0.9172	0.9198	1.0000
		10	3.5	5.6	0.9125	0.9199	1.0000
0.01	20	2	3.2	4.6	0.8423	0.9534	1.0000
		4	2.6	3.6	0.8677	0.9537	1.0000
		5	2.7	3.6	0.8675	0.9535	1.0000
0.01	30	3	4.4	5.9	0.8533	0.9540	1.0000
		5	3.9	5.1	0.8687	0.9549	1.0000
		6	4.0	5.2	0.8680	0.9550	1.0000
		10	4.5	6.2	0.8493	0.9516	1.0000
0.01	40	4	5.7	7.0	0.8564	0.9561	1.0000
		5	5.5	6.6	0.8630	0.9558	1.0000
		8	5.6	6.9	0.8603	0.9516	1.0000
		10	6.0	7.5	0.8511	0.9516	1.0000
0.02	20	2	5.0	5.8	0.7500	0.9559	1.0000
		4	4.1	4.6	0.7942	0.9553	1.0000
		5	4.1	4.7	0.7927	0.9555	1.0000
0.02	30	3	7.1	7.0	0.7621	0.9580	1.0000
		5	6.4	6.3	0.7851	0.9579	1.0000
		6	6.5	6.5	0.7826	0.9585	1.0000

		10	7.6	8.0	0.7461	0.9528	1.0000
0.02	40	4	9.4	8.0	0.7644	0.9601	1.0000
		5	9.1	7.8	0.7732	0.9596	1.0000
		8	9.4	8.5	0.7638	0.9523	1.0000
		10	10.2	9.4	0.7448	0.9525	1.0000
0.05	20	2	9.2	6.4	0.5419	0.9616	1.0000
		4	7.7	5.6	0.6127	0.9613	1.0000
		5	7.9	5.9	0.6051	0.9610	1.0000
0.05	30	3	12.9	6.9	0.5707	0.9642	1.0000
		5	12.2	7.1	0.5923	0.9642	1.0000
		6	12.6	7.6	0.5801	0.9640	1.0000
		10	14.9	9.5	0.5032	0.9568	1.0000
0.05	40	4	16.8	7.6	0.5797	0.9657	1.0000
		5	16.7	7.9	0.5823	0.9659	1.0000
		8	18.3	9.6	0.5436	0.9555	1.0000
		10	19.9	10.8	0.5021	0.9566	1.0000
0.07	20	2	11.1	6.0	0.4428	0.9636	1.0000
		4	9.7	5.7	0.5175	0.9634	1.0000
		5	9.9	6.0	0.5036	0.9634	1.0000
0.07	30	3	15.5	6.3	0.4848	0.9662	1.0000
		5	15.1	7.1	0.4954	0.9660	1.0000
		6	15.7	7.6	0.4762	0.9663	1.0000

0.07	30	10	18.6	9.4	0.3813	0.9589	1.0000
		4	20.2	7.1	0.4958	0.9673	1.0000
		5	20.4	7.8	0.4910	0.9675	1.0000
		8	22.7	9.7	0.4331	0.9574	1.0000
		10	24.6	10.7	0.3840	0.9587	1.0000
0.1	20	2	13.3	5.3	0.3328	0.9658	1.0000
		4	12.0	5.5	0.3985	0.9658	1.0000
		5	12.4	5.9	0.3776	0.9657	1.0000
0.1	30	3	18.4	5.6	0.3869	0.9676	1.0000
		5	18.7	6.9	0.3773	0.9677	1.0000
		6	19.5	7.4	0.3511	0.9675	1.0000
		10	22.8	8.9	0.2416	0.9615	0.9999
0.1	40	4	24.2	6.8	0.3951	0.9681	1.0000
		5	24.8	7.7	0.3795	0.9681	1.0000
		8	27.9	9.4	0.3028	0.9598	1.0000
		10	30.1	10.2	0.2470	0.9613	0.9999
0.15	20	2	15.9	4.3	0.2041	0.9676	1.0000
		4	15.1	5.2	0.2457	0.9676	1.0000
		5	15.7	5.6	0.2164	0.9677	0.9999
0.15	30	3	22.1	5.3	0.2621	0.9684	1.0000
		5	23.2	6.6	0.2260	0.9682	1.0000
		6	24.2	7.0	0.1934	0.9685	0.9999

		10	27.3	7.6	0.0888	0.9643	0.9999
0.15	40	4	29.6	7.0	0.2601	0.9685	1.0000
		5	30.7	7.7	0.2333	0.9684	1.0000
		8	34.1	8.7	0.1469	0.9626	0.9999
		10	36.1	8.9	0.0965	0.9642	0.9999
0.2	20	2	17.8	3.9	0.1093	0.9680	1.0000
		4	17.4	4.9	0.1281	0.9683	1.0000
		5	18.1	5.2	0.0972	0.9680	0.9999
0.2	30	3	25.2	5.4	0.1597	0.9685	1.0000
		5	26.7	6.3	0.1112	0.9683	0.9999
		6	27.6	6.5	0.0797	0.9685	0.9999
		10	30.0	6.4	-0.0011	0.9661	0.9999
0.2	40	4	34.0	7.0	0.1495	0.9687	1.0000
		5	35.2	7.5	0.1190	0.9684	0.9999
		8	38.3	7.9	0.0433	0.9646	0.9999
		10	39.7	7.6	0.0074	0.9660	0.9999

Figure S1. The process of 3-stage pooled-plasma HCV RNA testing

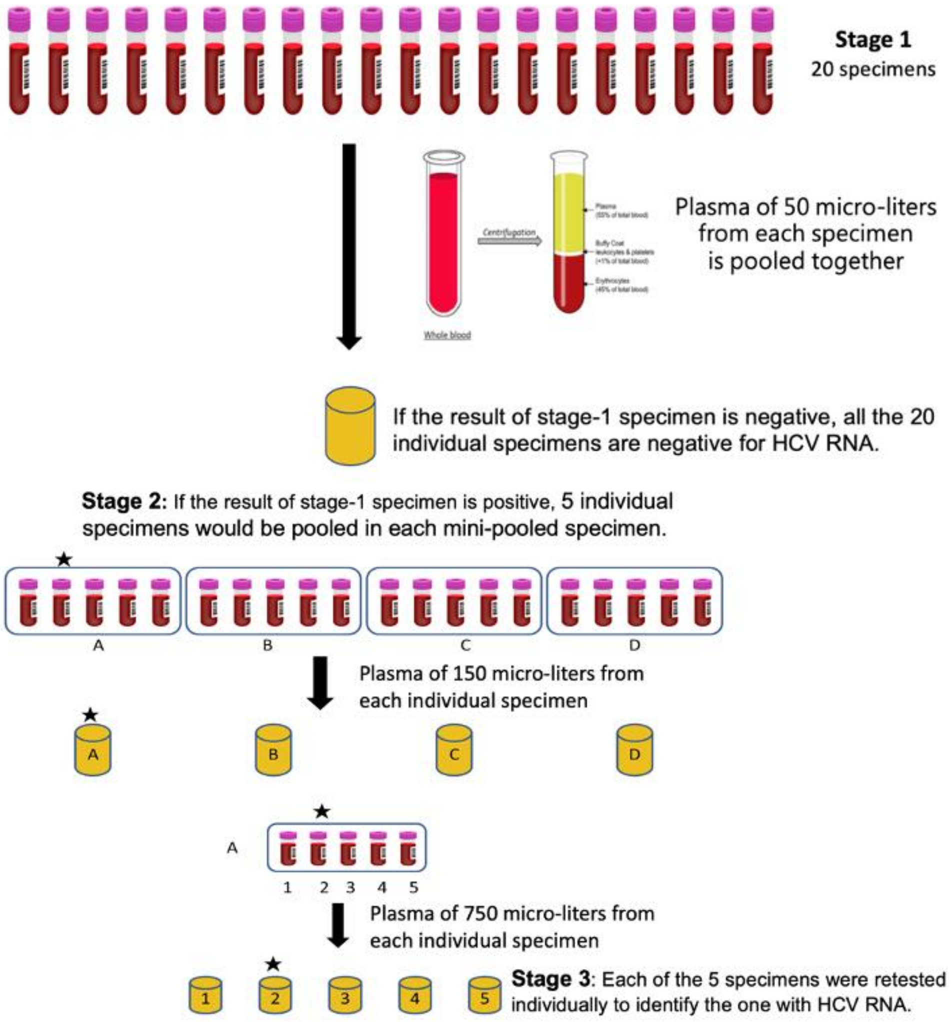
Stage 1
20 specimens
Centrifugation
Whole blood
Plasma of 50 micro-liters
from each specimen
is pooled together
If the result of stage-1 specimen is negative, all the 20 individual specimens are negative for HCV RNA.
Stage 2: If the result of stage-1 specimen is positive, 5 individual specimens would be pooled in each mini-pooled specimen.
A
B
C
D
Plasma of 150 micro-liters from each individual specimen
A
B
C
D
A
1 2 3 4 5
Plasma of 750 micro-liters from each individual specimen
1
2
3
4
5
Stage 3: Each of the 5 specimens were retested individually to identify the one with HCV RNA.

Footnote: The star sign indicates the individual specimen positive for HCV RNA.

Figure S2. Outcomes of 62 people living with HIV in whom HCV viremia were identified by three-stage pooled-plasma HCV testing in the present study

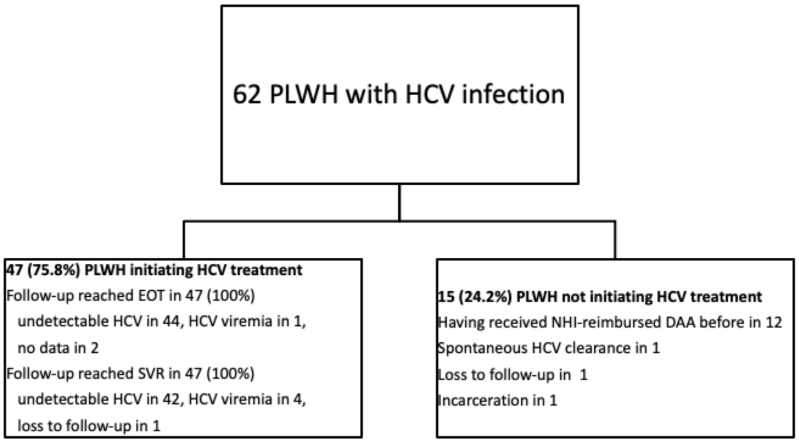

Abbreviations: EOT, end of treatment; HCV, hepatitis C virus; NHI, National Health Insurance; PLWH, people living with HIV; SVR12, sustained virologic response 12 weeks off-therapy

Figure S3. Pooled sensitivity against prevalence with individual sensitivity of 98.94% and individual specificity of 99.99% (Given the detection limit of 15 IU/mL, the value of the undetectable individual specimen is assigned to 0 IU/mL in the left panel, and that of the undetectable individual specimen is assigned to 14 IU/mL in the right panel). The lower the HCV prevalence or larger pooled size, the lower sensitivity of pooled HCV RNA testing. With the HCV viremia prevalence of 1% and the undetectable values of 14 IU/mL, the pooled sensitivity decreased to around 97% while the individual sensitivity was 99.98%. If undetectable values were assigned to be 0 IU/mL, the pooled sensitivity decreased to only around 92%. However, if undetectable values were set as 0 IU/mL, the pooled sensitivity for N=20 was much greater than those for N=30 and N=40, quite different from the conditions with the undetectable value of 14 IU/mL.

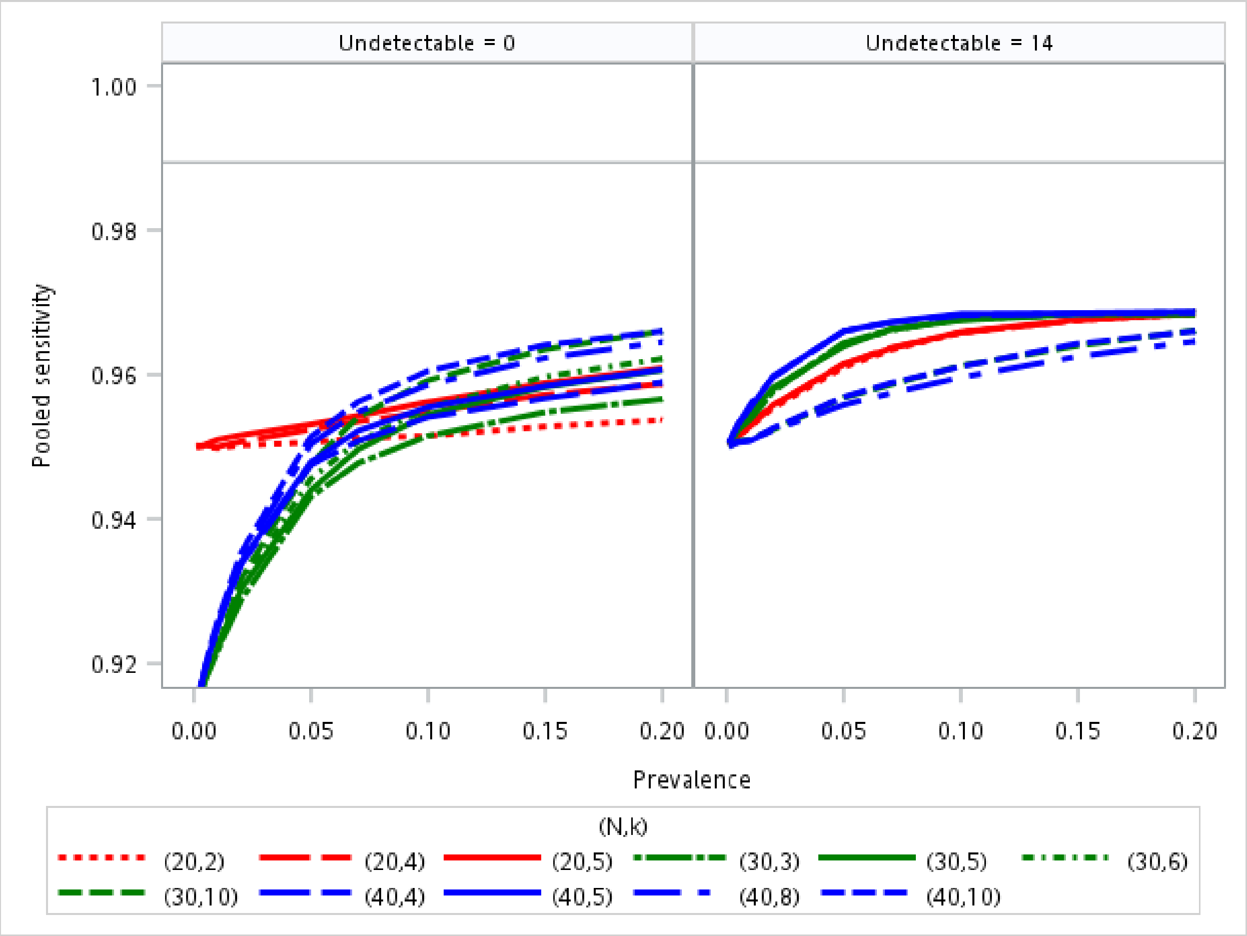
Undetectable = 0
Undetectable = 14
Pooled sensitivity
1.00
0.98
0.96
0.94
0.92
0.00
0.05
0.10
0.15
0.20
Prevalence
(N,k)
(20,2)
(20,4)
(20,5)
(30,3)
(30,5)
(30,6)
(30,10)
(40,4)
(40,5)
(40,8)
(40,10)

Figure S4. Pooled specificity against prevalence with individual sensitivity of 98.94% and individual specificity of 99.99% (Given the detection limit of 15 IU/mL, the value of the undetectable individual specimen is assigned to 0 IU/mL in the left panel, and that of the undetectable individual specimen is assigned to 14 IU/mL in the right panel). For the pooled specificity, all were better than the individual specificity regardless of any HCV viremia prevalence.

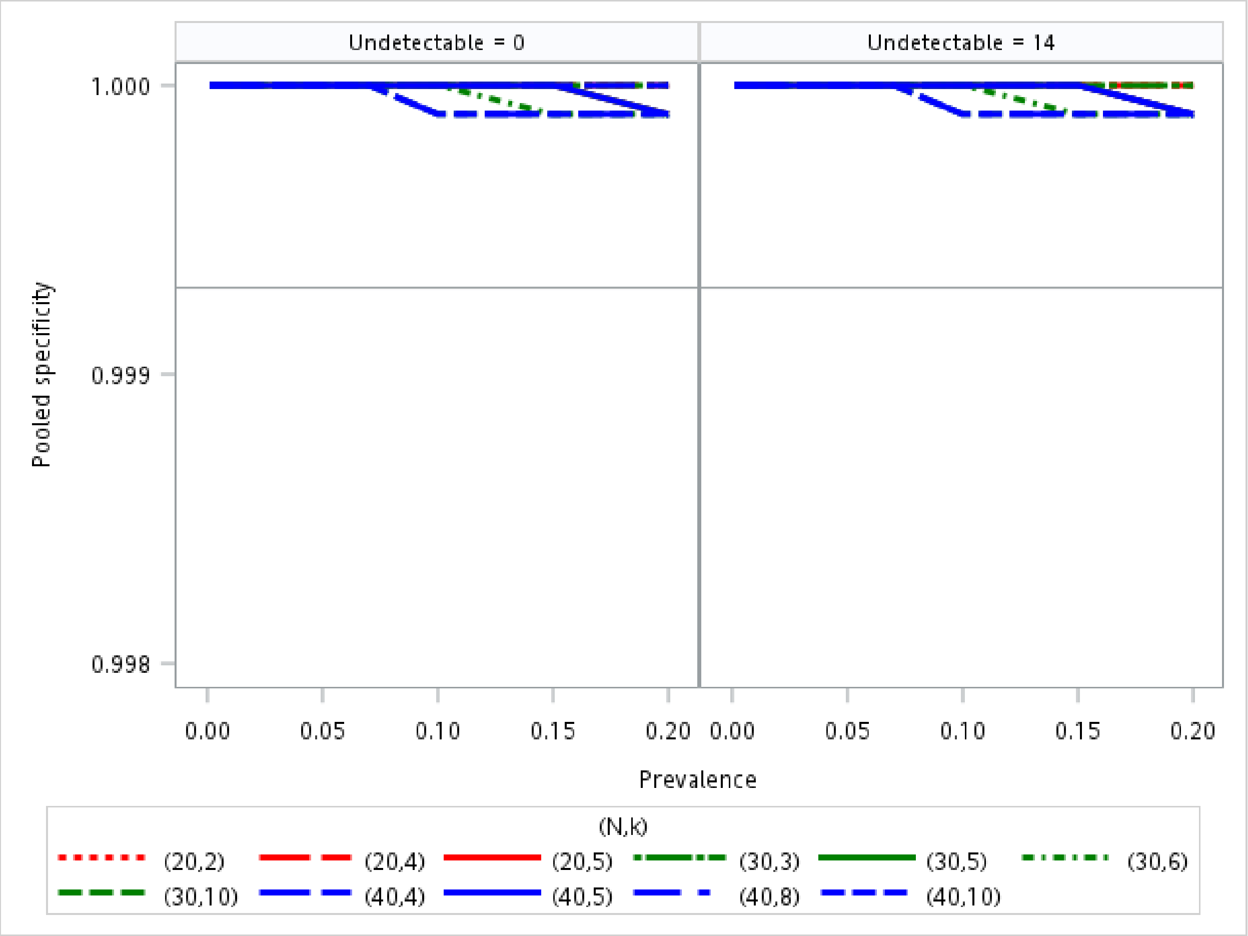

Figure S5. Cost reduction against prevalence with different lower individual sensitivities (I_Se) and individual specificities (I_Sp) in the setting of the combinations of pooled sizes of (pool size, N = 20, 30, or 40; mini-pool size, k=5) and the value of undetectable individual specimen of 14 IU/mL. The performance remained similar in all settings.

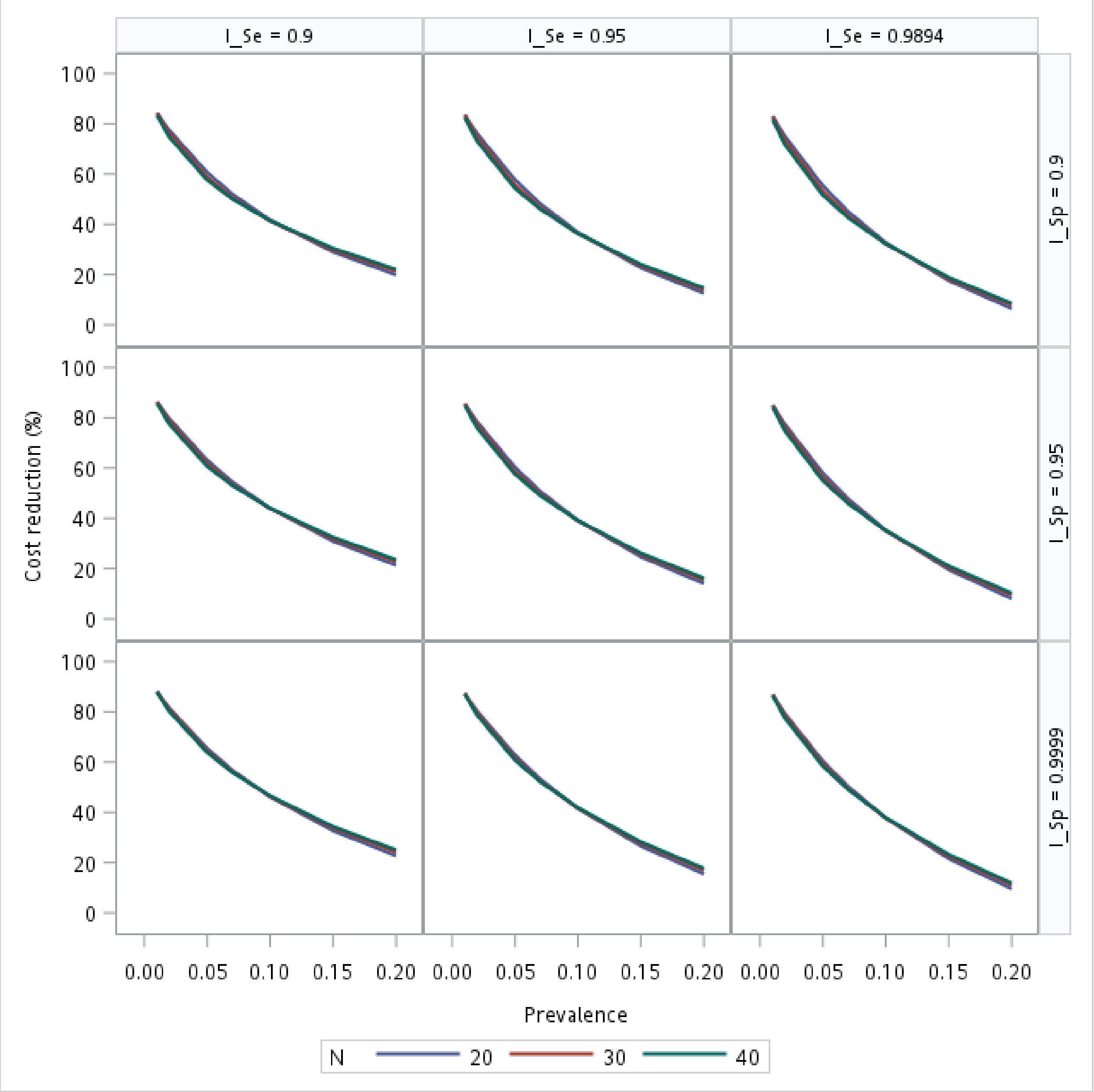

Figure S6. Pooled sensitivity against prevalence with different individual sensitivities (I_Se) and individual specificities (I_Sp) in the setting of the combinations of pooled sizes of (pool size, N = 20, 30, or 40; mini-pool size, k=5) and the value of undetectable individual specimen of 14 IU/mL. When the individual sensitivity was 90%, the pooled sensitivity was only around 72%, which was considered poor performance.

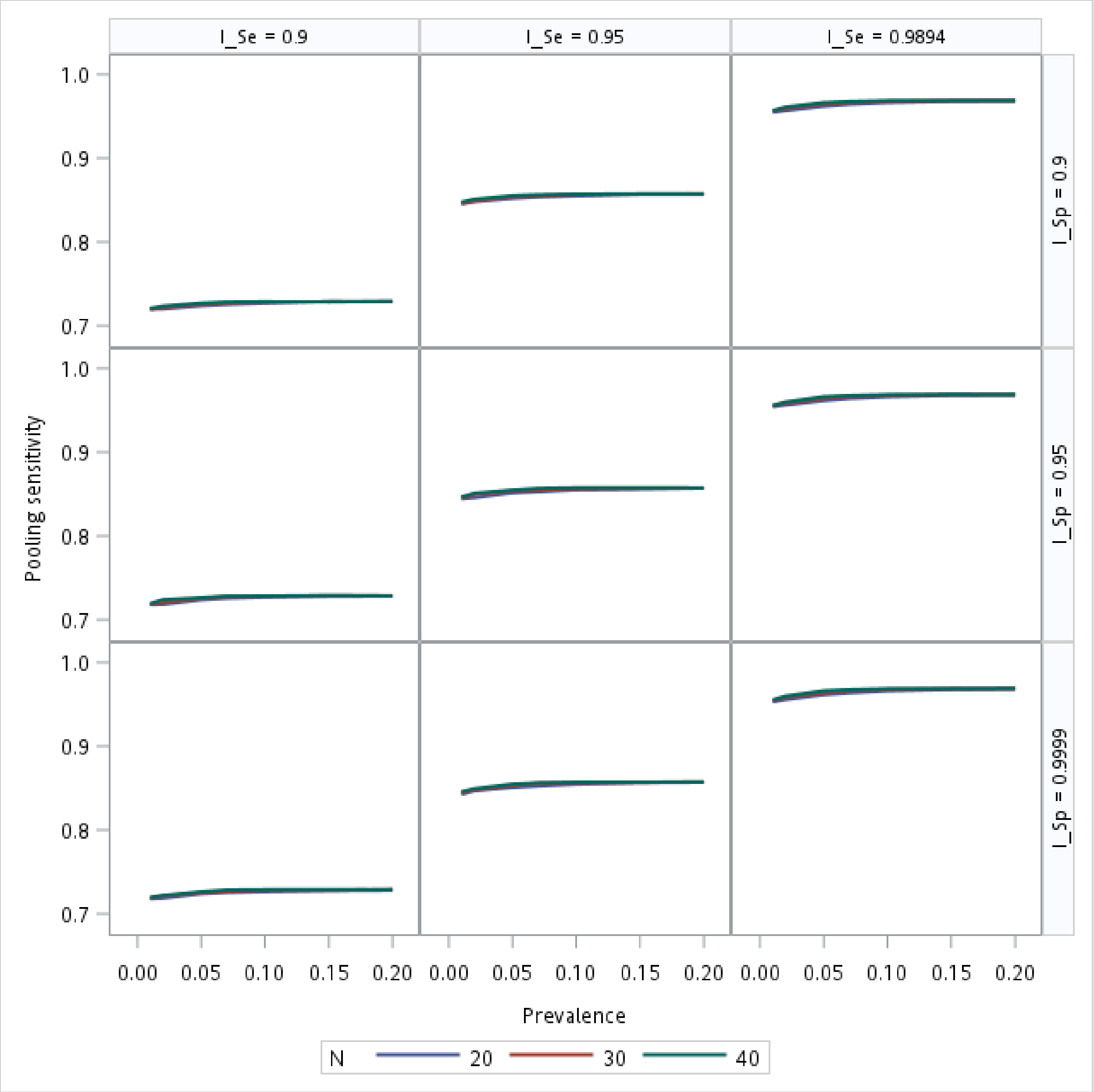

Figure S7. Pooled specificity against prevalence with different individual sensitivities (I_Se) and individual specificities (I_Sp) in the setting of the combinations of pooled sizes of (pool size, N = 20, 30, or 40; mini-pool size, k=5) and the value of undetectable individual specimen of 14 IU/mL. The pooled specificity decreased with a decrease of individual specificity, but the decrease was acceptable since it was still around 94% even when the HCV viremia prevalence was 20%.

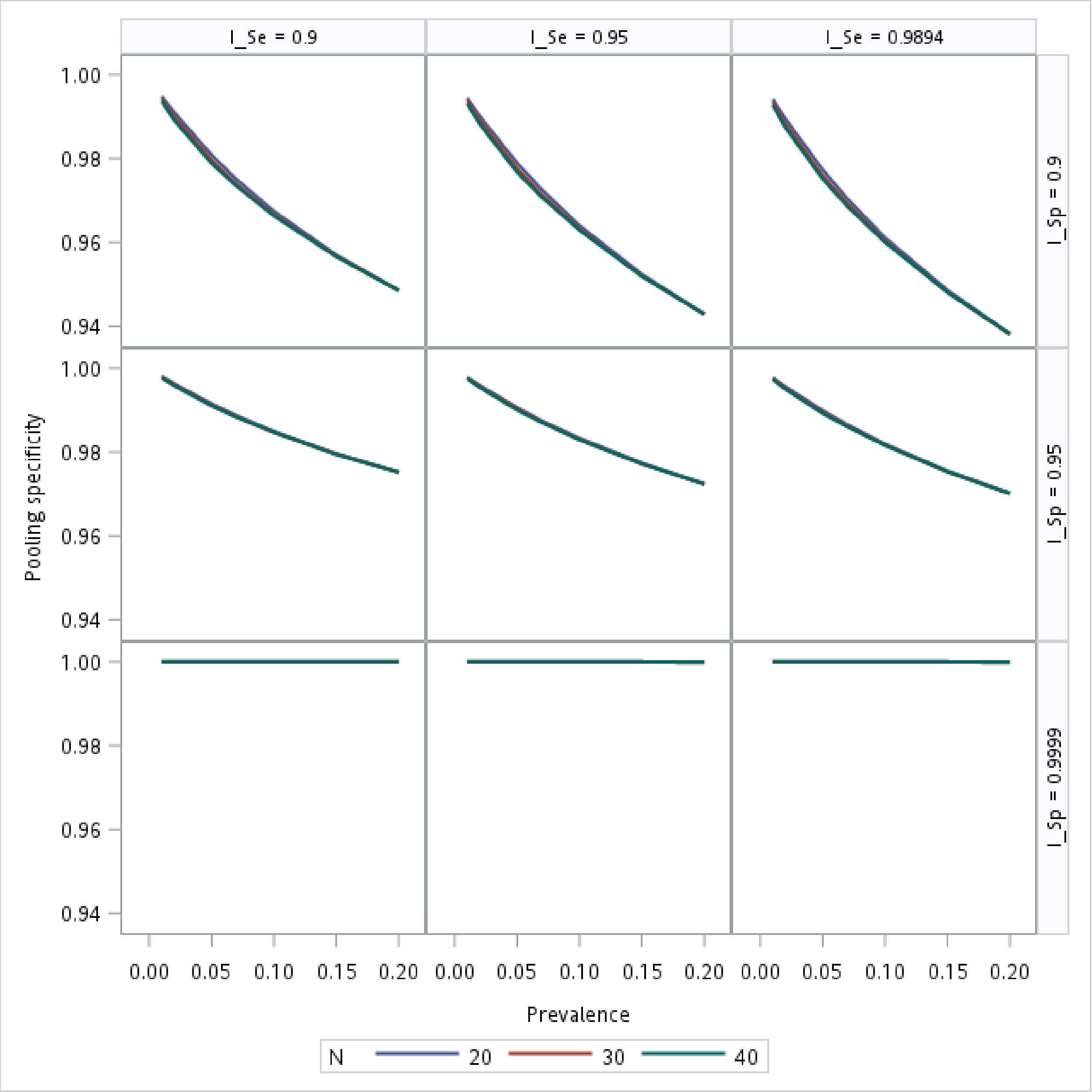

Supplement: SUPPLEMENTAL FILE 1 — Supplemental material. Download spectrum.02437-21-s001.pdf, PDF file, 0.6 MB [file spectrum.02437-21-s001.pdf]
